# Serum progesterone distribution in normal pregnancies compared to pregnancies complicated by threatened miscarriage from 5 to 13 weeks gestation: a prospective cohort study

**DOI:** 10.1186/s12884-018-2002-z

**Published:** 2018-09-05

**Authors:** Chee Wai Ku, John C. Allen Jr, Sze Min Lek, Ming Li Chia, Nguan Soon Tan, Thiam Chye Tan

**Affiliations:** 1 Department of Obstetrics and Gynecology, KK Women’s and Children’s Hospital, Singapore, 100 Bukit Timah Road, Singapore, 229899 Singapore; 2 Duke-National University of Singapore Graduate Medical School, Singapore, Singapore; 3 Lee Kong Chian School of Medicine, Nanyang Technological University, Singapore, Singapore; 4 School of Biological Sciences, Nanyang Technological University, Singapore, Singapore; 5 Institute of Molecular and Cell Biology, A*STAR, Singapore, Singapore

**Keywords:** Serum progesterone, First trimester distribution, Progesterone nomogram, Threatened miscarriage

## Abstract

**Background:**

Progesterone is a critical hormone in early pregnancy. A low level of serum progesterone is associated with threatened miscarriage. We aim to establish the distribution of maternal serum progesterone in normal pregnancies compared to pregnancies complicated by threatened miscarriage from 5 to 13 weeks gestation.

**Methods:**

This is a single centre, prospective cohort study of 929 patients. Women from the Normal Pregnancy [NP] cohort were recruited from antenatal clinics, and those in the Threatened Miscarriage [TM] cohort were recruited from emergency walk-in clinics. Women with multiple gestations, missed, incomplete or inevitable miscarriage were excluded from the study. Quantile regression was used to characterize serum progesterone levels in the NP and TM cohorts by estimating the 10th, 50th and 90th percentiles from 5 to 13 weeks gestation. Pregnancy outcome was determined at 16 weeks of gestation. Subgroup analysis within the TM group compared progesterone levels of women who subsequently miscarried with those who had ongoing pregnancies at 16 weeks of gestation.

**Results:**

Median serum progesterone concentration demonstrated a linearly increasing trend from 57.5 nmol/L to 80.8 nmol/L from 5 to 13 weeks gestation in the NP cohort. In the TM cohort, median serum progesterone concentration increased from 41.7 nmol/L to 78.1 nmol/L. However, median progesterone levels were uniformly lower in the TM cohort by approximately 10 nmol/L at every gestation week. In the subgroup analysis, median serum progesterone concentration in women with ongoing pregnancy at 16 weeks gestation demonstrated a linearly increasing trend from 5 to 13 weeks gestation. There was a marginal and non-significant increase in serum progesterone from 19.0 to 30.3 nmol/L from 5 to 13 weeks gestation in women who eventually had a spontaneous miscarriage.

**Conclusions:**

Serum progesterone concentration increased linearly with gestational age from 5 to 13 weeks in women with normal pregnancies. Women with spontaneous miscarriage showed a marginal and non-significant increase in serum progesterone. This study highlights the pivotal role of progesterone in supporting an early pregnancy, with lower serum progesterone associated with threatened miscarriage and a subsequent complete miscarriage at 16 weeks gestation.

**Electronic supplementary material:**

The online version of this article (10.1186/s12884-018-2002-z) contains supplementary material, which is available to authorized users.

## Background

Threatened miscarriage is defined as vaginal bleeding with or without abdominal pain and a closed cervical os in early pregnancy. It affects 15 - 20% of all pregnancies [1, 2] and is a risk factor for adverse pregnancy outcomes including preeclampsia, pre-term delivery, intrauterine growth restriction, preterm premature rupture of membranes and placental abruption [3]. Amongst women with threatened miscarriage, 15 – 25% progress to spontaneous miscarriage [4] and they are 2.6 times more likely to miscarry as compared to pregnant women with no bleeding [5, 6]. Women with threatened miscarriage are often extremely anxious about the pregnancy outcome, and this is not aided by the lack of predictive models that prognosticate and triage such women into the high or low risk of miscarriage [4].

Progesterone is a critical hormone during implantation. It sustains decidualization [7], controls uterine contractility and promotes maternal immune tolerance to the fetal semi-allograft [8]. Lymphocytes, in the presence of progesterone, also release progesterone-induced blocking factor (PIBF). PIBF is a pivotal mediator in progesterone-dependent immunomodulation [9, 10] and has a regulatory role in anti-fetal immune responses during pregnancy [11]. One of the earliest studies on progesterone in pregnancy showed an increasing trend of plasma progesterone from conception to delivery [12]. A more recent study by Schock *et al* further highlighted this increasing trend throughout pregnancy [13]. However, little is known about the distribution of serum progesterone in early pregnancy.

Many studies have shown that low serum progesterone is associated with threatened miscarriage. Our group has validated a single serum progesterone cutoff of 35 nmol/L taken at presentation with a threatened miscarriage can differentiate women at high or low risk of subsequent miscarriage [14, 15]. Hence, women with normal pregnancies (low risk) with no bleeding may have a different serum progesterone distribution compared to women with threatened miscarriage. In this study, we aim to establish the distribution of maternal serum progesterone in normal pregnancies and pregnancies complicated by threatened miscarriage from 5 to 13 weeks’ gestation.

## Methods

A total of 929 pregnant women, aged 21 years and above, presenting at the KK Women’s and Children’s Hospital (KKH) antenatal clinics and 24-hour Women’s Clinic from January 2013 to December 2016 were recruited. Inclusion criteria were a single intrauterine pregnancy between gestation weeks 5 to 13 (confirmed and dated by ultrasonography), with pregnancy-related per vagina bleeding were recruited in the Threatened Miscarriage [TM] cohort (*n* = 479) while those with no pregnancy-related per vagina bleeding were recruited in the low risk of miscarriage (normal pregnancy [NP]) cohort (*n* = 450). Women with multiple gestations, previous episodes of per vagina bleeding or those treated with progesterone for previous per vagina bleeding in the current pregnancy, or women diagnosed with an inevitable miscarriage, missed miscarriage, blighted ovum or planned termination of pregnancy were excluded.

Maternal blood samples were taken to measure serum progesterone level at presentation as previously described [15]. Blood was collected in plain tubes and centrifuged for 10 min at 3000 *g* within 2 hours of collection. Serum progesterone level was measured in the KKH clinical laboratory using a commercial ARCHITECT progesterone kit (Abbott, Ireland).

Covariates for the analysis were maternal demographics, health, obstetric and lifestyle factors collected by an investigator administered the questionnaire in either English or Chinese (Table 1).

**Table 1:** Serum progesterone and maternal characteristics at baseline, for low risk and high risk women with threatened miscarriage

Variable	Normal Pregnancy [NP] Cohort (*n*=450)	Threatened Miscarriage [TM] Cohort (*n*=479)	*p*-value
Demographics			
Maternal Age (yr)	30.9 (4.0)^a^	30.6 (4.5)	0.058
Gestational Age (wk)	8.4 (2.1)	7.3 (1.4)	<0.0001
Body mass index	22.9 (4.2)	23.1 (4.6)	0.459
Previous Miscarriage (%)	18.7	23.0	0.107
Diabetes mellitus (%)	0.21	0.23	1.000
Smoking (%)	2.93	4.59	0.322
Outcome measures			
Serum Progesterone (nmol/L)	71.8 (27.2)	53.6 (25.2)	<0.0001
Miscarriage rate (%)	5.4	21.5	<0.0001

^a^Mean (SD)

### Outcome measures and follow-up

The primary outcome measured was a spontaneous miscarriage, defined by self-reported uterine evacuation after an inevitable or incomplete miscarriage, or complete miscarriage with an empty uterus, by the 16^th^ week of gestation. All participants were contacted at the 16^th^ week of pregnancy to verify pregnancy status.

### Statistical Methods

Baseline maternal demographics and pregnancy characteristics were statistically compared between two study cohorts: (i) patients with no pregnancy-related per vagina bleeding [NP] and (ii) patients with pregnancy-related per vagina bleeding [TM]. The 2-sample t-test was used to compare continuous baseline variables and Fisher’s exact test to compare categorical variables.

Quantile regression was used to characterize serum progesterone levels in the NP and TM cohorts by estimating the 10th, 50th and 90th percentiles from 5 to 13 weeks gestation. Pregnancy outcome was determined at 16 weeks of gestation. Subgroup analysis was carried out within the TM cohort to compare progesterone levels of women who experienced spontaneous miscarriage [TMM] with those who had ongoing pregnancies at 16 weeks of gestation [TMO]. The numbers of patients that presented in each gestation week in the different groups (NP, TM, TMM and TMO) were summarized in Additional file 1: Table S1 and Additional file 2: Figure S1).

This study is funded by the Ministry of Health Industry Alignment Fund Category 1 research fund.

## Results

Miscarriage rates were significantly lower in the normal pregnancy (low risk) [NP] cohort (5.4%) compared to those who presented with a threatened miscarriage (21.5%) (*P* < 0.0001). Mean serum progesterone was significantly higher in the NP cohort (71.8 ± 27.2 nmol/L) compared to those in the threatened miscarriage [TM] cohort (53.6 ± 25.2 nmol/L) (*P* < 0.0001). Women in the NP cohort tend to present later for their booking visit (8.4 ± 2.1 weeks vs 7.3 ± 1.4 weeks) (*P* < 0.0001). There were no differences in maternal age, body mass index (BMI), history of previous miscarriages and smoking, or having comorbidities such as diabetes mellitus (Table 1).

**Fig. 1:**
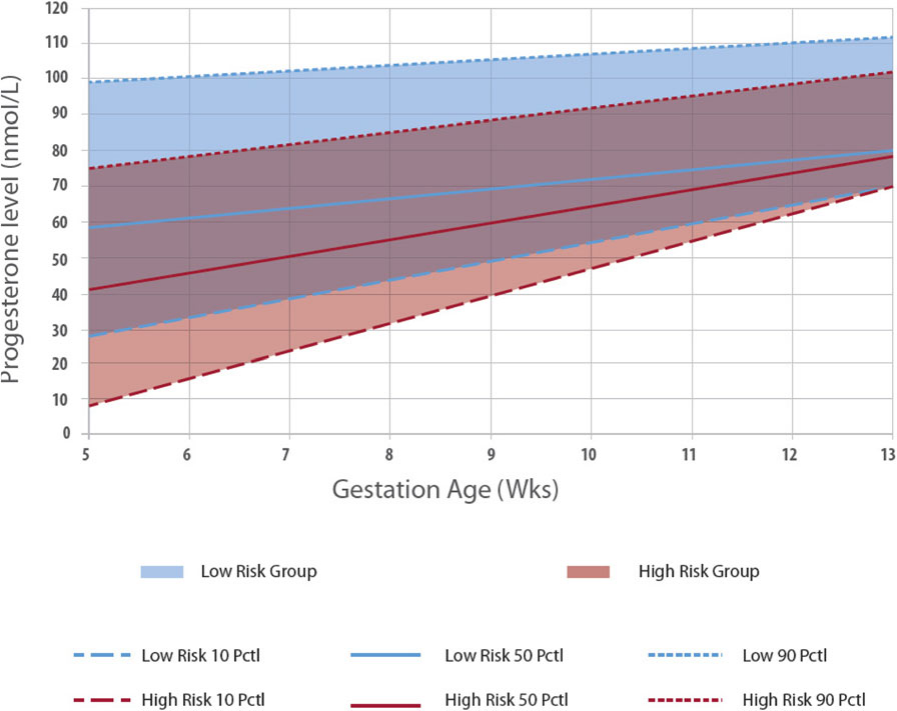
Distribution of serum progesterone across gestation weeks 5-13 amongst women with normal pregnancy [NP] vs threatened miscarriage [TM]

Serum progesterone concentration demonstrated a linearly increasing trend from 57.5 nmol/L to 80.8 nmol/L from 5 to 13 weeks gestation in the NP cohort, with a median trend gradient of b_NP_ = 2.91 (*p* = 0.0020) (Fig. 1, Additional file 1: Table S1A and Additional file 2: Figure S1A). In the TM cohort, serum progesterone concentration increased from 41.7 nmol/L to 78.1 nmol/L from 5 to 13 weeks gestation, with a trend gradient of b_TM_ = 4.55 (*p* <0.0001) (Fig. 1, Additional file 1: Table S1B and Additional file 2: Figure S1B). Median progesterone levels were uniformly lower in the TM cohort by approximately 10 nmol/L, converging towards the end of the first trimester with similar values at 13 weeks gestation (Fig. 1).

**Fig. 2:**
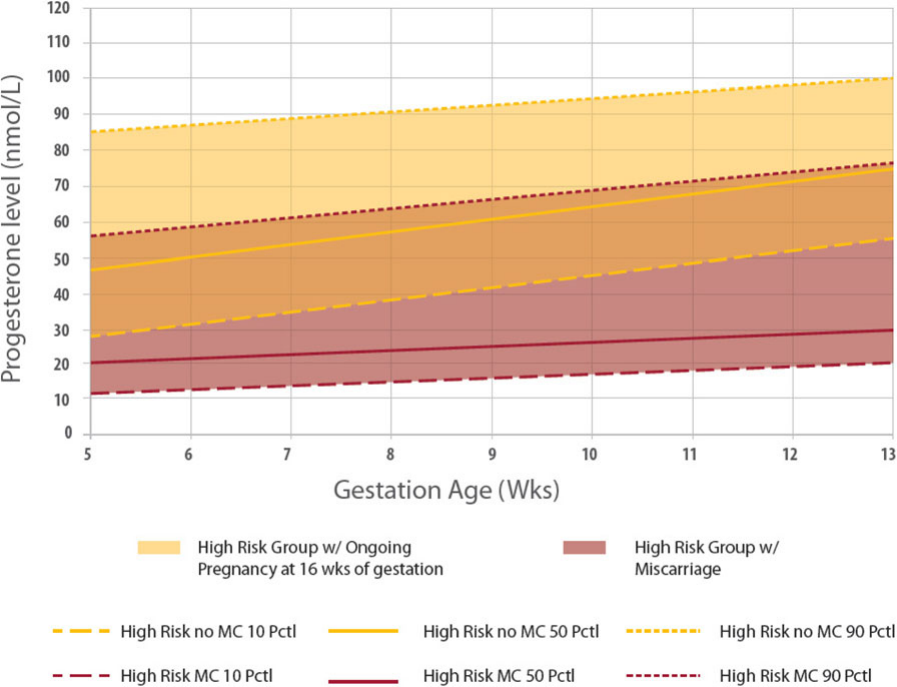
Threatened miscarriage [TM] subgroup analysis of women with ongoing pregnancy vs spontaneous miscarriage

In the subgroup analysis, women in the TM cohort were divided into those with ongoing pregnancies at 16 weeks gestation [TMO] compared to those who experienced spontaneous miscarriage before or at 16 weeks gestation [TMM]. Serum progesterone levels in women with ongoing pregnancy at 16 weeks gestation demonstrated a linearly increasing trend from 47.4 nmol/L to 75.0 nmol/L from 5 to 13 weeks gestation, with a trend gradient of b_TMO_ = 3.45 (*p* <0.0001) (Fig. 2, Additional file 1: Table S1C and Additional file 2: Figure S1C). Comparatively, there was a non-significant and marginal increase in serum progesterone from 19.0 nmol/L to 30.3 nmol/L from 5 to 13 weeks gestation in women who eventually experienced spontaneous miscarriage before or at 16 weeks gestation, with a trend gradient of b_TMM_ = 1.41 (*p* = 0.710) (Fig. 2, Additional file 1: Table S1D and Additional file 2: Figure S1D) .

## Discussion

### Main Findings

Miscarriage rates were significantly lower in the normal pregnancy (low risk) [NP] cohort (5.4%) compared to those in the threatened miscarriage [TM] cohort. Mean serum progesterone was significantly higher in the NP cohort compared to those in the TM cohort. Serum progesterone increased linearly with gestational age from 5 to 13 weeks in women with normal pregnancies. Women with spontaneous miscarriage showed a marginal and non-significant increase in serum progesterone.

### Strengths and Limitations

This is one of the first prospective cohort studies describing the distribution of serum progesterone in normal pregnancies (low risk) compared to pregnancies that is complicated by threatened miscarriage. There are several limitations of this study. Specifically, the mean gestation at presentation for women in the normal pregnancy cohort is 8.4, while that for women in the threatened miscarriage cohort is 7.3. Women with low risk pregnancies tend to present later, whereas those with bleeding in early pregnancy will seek medical attention promptly. This may be a potential confounder accounting for the higher mean serum progesterone in the normal pregnancy cohort. In addition, the distribution of serum progesterone across gestations is not taken from the same patient, so it may be affected by inherent biological variation amongst patients. Further studies need to be conducted to evaluate the underlying pathophysiology of low progesterone and miscarriage, and examine the role of progestogens in the management of women with threatened miscarriage.

### Interpretation

Many studies have shown that low serum progesterone is associated with poor pregnancy outcomes [16, 17], and our results lend further weight to the pivotal role of progesterone in early pregnancy. In the NP cohort, serum progesterone increased linearly with gestation age from 5 to 13 weeks, with a similar trend observed in TM cohort who had an ongoing pregnancy at 16 weeks gestation.

Progesterone is secreted by the corpus luteum, which only lasts for 14 days if a pregnancy did not occur. In early pregnancy, beta human chorionic gonadotropin (βhCG) secreted by syncytiotrophoblasts maintains the corpus luteum, which allows it to continue secreting progesterone until the placenta takes over its function at 7 to 9 weeks of gestation. Progesterone causes secretory changes in the endometrium of the uterus and is essential for successful implantation of the embryo [18]. Following implantation, elevated levels of circulating progesterone secreted by the placenta acting through progesterone receptors maintain uterine quiescence [19] and stimulate morphological changes to the cervix and other tissues that help to maintain pregnancy [20].

Luteal phase deficiency (LPD) is a condition of insufficient progesterone to maintain a normal secretory endometrium and allow for normal embryo implantation and growth [21]. This is one of many etiologies associated with early pregnancy loss [22]. Two mechanisms have been proposed that results in LPD. The first and likely more common cause relates to the impaired function of the corpus luteum resulting in insufficient progesterone and estradiol secretion [23]. The impaired function can be the result of improper development of the dominant follicle destined to become the corpus luteum or aberrant stimulation of a normally developed follicle, leading to deficiencies in progesterone production. The second mechanism suggests an inability of the endometrium to mount a proper response to appropriate estradiol and progesterone exposure [24].

Apart from LPD, there are other proposed causes of spontaneous miscarriage. More than half of clinically recognized pregnancy loss have been attributed to chromosomal abnormalities [25, 26]. Chromosomal abnormalities could be associated with changes in progesterone levels [27]. Progesterone was shown to be lower in pregnancies with trisomy 13 and trisomy 18 [28]. Other causes of spontaneous miscarriage include maternal factors such as infections and maternal disease states [29].

In women with threatened miscarriage, serum progesterone concentration also increased linearly with gestation, but exhibited a downward displacement of the graph with lower median progesterone levels at every gestation week compared to the low risk group, converging towards the end of the first trimester with similar values at 13 weeks gestation. In women with ongoing pregnancies, vaginal bleeding may be due to disruption of decidual vessels at the maternal-fetal interface [30].

In the subgroup analysis of women with threatened miscarriage, those who experienced a spontaneous miscarriage at or before 16 weeks gestation have a lower serum progesterone level. Many prior studies have shown that the mean serum progesterone level in non-viable gestations are low, ranging between 6.8 – 12 ng/ml (21.6 – 38.2 nmol/L) [31–33], but very few have described the distribution of progesterone in early pregnancy. Interestingly, we found that in women with spontaneous miscarriage at or before 16 weeks gestation, there was only a marginal increase in serum progesterone across gestations, with much lower serum progesterone levels between 20 nmol/L to 30 nmol/L. Unlike normal pregnancies, serum progesterone did not increase significantly regardless of gestation in women with spontaneous miscarriage.

## Conclusion

This study highlights the pivotal role of progesterone in supporting an early pregnancy, where lower serum progesterone is associated with threatened miscarriage and a subsequent complete miscarriage at 16 weeks gestation. This may serve as a platform for the development of reference ranges for women who present with low risk pregnancies or threatened miscarriage to predict the risk of subsequent spontaneous miscarriages based on their progesterone levels.

## Additional files

Additional file 1: **Table S1A**. Distribution of serum progesterone across gestation weeks 5 – 13 amongst women with low risk pregnancy [NP]. **Table S1B**. Distribution of serum progesterone across gestation weeks 5 – 13 amongst women with threatened miscarriage [TM]. **Table S1C**. Distribution of serum progesterone across gestation weeks 5 – 13 amongst women who presented with threatened miscarriage and had ongoing pregnancy at 16 weeks [TMO]. **Table S1D**. Distribution of serum progesterone across gestation weeks 5 – 13 amongst women who presented with threatened miscarriage and had a spontaneous miscarriage at or before 16 weeks [TMM]. (DOCX 20 kb)

Additional file 2: **Figure S1A**. Distribution of serum progesterone across gestation weeks 5 – 13 amongst women with low risk pregnancy [NP]. **Figure S1B**. Distribution of serum progesterone across gestation weeks 5 – 13 amongst women with threatened miscarriage [TM]. **Figure S1C**. Distribution of serum progesterone across gestation weeks 5 – 13 amongst women who presented with threatened miscarriage and had ongoing pregnancy at 16 weeks [TMO]. **Figure S1D**. Distribution of serum progesterone across gestation weeks 5 – 13 amongst women who presented with threatened miscarriage and had a spontaneous miscarriage at or before 16 weeks [TMM]. (ZIP 141 kb)

## Abbreviations

NP: Normal pregnancy
TM: Threatened miscarriage
TMO: Threatened miscarriage with ongoing pregnancies at 16 weeks gestation
TMM: Threatened miscarriage with spontaneous miscarriage before or at 16 weeks gestation
KKH: KK Women’s and Children’s Hospital
BMI: Body mass index
βhCG: Beta human chorionic gonadotropin
LPD: Luteal phase deficiency
DM: Diabetes mellitus
