# Supplementary material for: Serum progesterone distribution in normal pregnancies compared to pregnancies complicated by threatened miscarriage from 5 to 13 weeks gestation: a prospective cohort study
Source: BMC Pregnancy Childbirth. 2018 Sep 5;18:360. doi: 10.1186/s12884-018-2002-z (PMC6126027; doi:10.1186/s12884-018-2002-z)
Supplement: Supplementary file 1 — Table S1A. Distribution of serum progesterone across gestation weeks 5 – 13 amongst women with low risk pregnancy [NP]. Table S1B. Distribution of serum progesterone across gestation weeks 5 – 13 amongst women with threatened miscarriage [TM]. Table S1C. Distribution of serum progesterone across gestation weeks 5 – 13 amongst women who presented with threatened miscarriage and had ongoing pregnancy at 16 weeks [TMO]. Table S1D. Distribution of serum progesterone across gestation weeks 5 – 13 amongst women who presented with threatened miscarriage and had a spontaneous miscarriage at or before 16 weeks [TMM]. (DOCX 20 kb) [file 12884_2018_2002_MOESM1_ESM.docx]

| **GA** | **n** | **Mean** | **SD** | **Min** | **10^th^ %** | **Median** | **90^th^ %** | **Max** |
| --- | --- | --- | --- | --- | --- | --- | --- | --- |
| **5** | **29** | 69.37 | 33.73 | 10.65 | 29.24 | 63.55 | 126.84 | 144.56 |
| **6** | **31** | 57.25 | 20.44 | 18.61 | 32.07 | 52.58 | 78.54 | 109.98 |
| **7** | **27** | 62.47 | 20.14 | 29.76 | 34.22 | 62.28 | 89.34 | 103.51 |
| **8** | **35** | 69.85 | 23.35 | 35.36 | 42.87 | 66.73 | 102.89 | 147.51 |
| **9** | **52** | 80.70 | 29.45 | 34.16 | 49.40 | 73.91 | 110.60 | 174.00 |
| **10** | **41** | 77.52 | 26.57 | 34.00 | 50.07 | 74.78 | 107.32 | 161.16 |
| **11** | **9** | 79.60 | 25.59 | 45.43 | 45.43 | 72.57 | 131.20 | 131.20 |
| **12** | **15** | 97.97 | 24.11 | 70.89 | 74.01 | 90.20 | 121.91 | 169.43 |

Table S1A. Distribution of serum progesterone across gestation weeks 5 – 13 amongst women with **low risk pregnancy [NP]**

GA: gestational age in weeks, n: number of patients, SD: standard deviation, Min: minimum value of serum progesterone in nmol/L, Max: maximum value of serum progesterone in nmol/L

Table S1B. Distribution of serum progesterone across gestation weeks 5 – 13 amongst women with **threatened miscarriage [TM]**

| **GA** | **n** | **Mean** | **SD** | **Min** | **10^th^ %** | **Median** | **90^th^ %** | **Max** |
| --- | --- | --- | --- | --- | --- | --- | --- | --- |
| **5** | **14** | 54.44 | 36.94 | 11.83 | 18.11 | 46.33 | 83.20 | 165.16 |
| **6** | **248** | 48.07 | 24.63 | 7.27 | 15.85 | 46.28 | 76.76 | 132.10 |
| **7** | **74** | 53.84 | 24.36 | 13.84 | 23.89 | 53.04 | 88.32 | 148.67 |
| **8** | **66** | 57.80 | 20.92 | 4.79 | 36.21 | 57.66 | 78.71 | 120.43 |
| **9** | **37** | 68.33 | 25.22 | 34.93 | 38.65 | 63.16 | 96.79 | 169.70 |
| **10** | **39** | 67.00 | 22.21 | 16.00 | 45.48 | 66.56 | 94.35 | 110.69 |
| **11** | **1** | 61.47 | . | 61.47 | 61.47 | 61.47 | 61.47 | 61.47 |

GA: gestational age in weeks, n: number of patients, SD: standard deviation, Min: minimum value of serum progesterone in nmol/L, Max: maximum value of serum progesterone in nmol/L

Table S1C. Distribution of serum progesterone across gestation weeks 5 – 13 amongst women who presented with **threatened miscarriage and had ongoing pregnancy at 16 weeks [TMO]**

| **GA** | **n** | **Mean** | **SD** | **Min** | **10^th^ %** | **Median** | **90^th^ %** | **Max** |
| --- | --- | --- | --- | --- | --- | --- | --- | --- |
| **5** | **10** | 63.85 | 38.69 | 32.94 | 36.36 | 47.87 | 124.18 | 165.16 |
| **6** | **170** | 56.02 | 22.04 | 9.80 | 31.11 | 53.17 | 82.43 | 132.10 |
| **7** | **60** | 58.40 | 23.62 | 17.43 | 33.67 | 53.90 | 91.35 | 148.67 |
| **8** | **56** | 61.30 | 18.70 | 24.57 | 38.66 | 59.17 | 86.36 | 120.43 |
| **9** | **33** | 69.72 | 25.85 | 36.33 | 47.80 | 64.13 | 96.79 | 169.70 |
| **10** | **35** | 71.26 | 17.45 | 45.48 | 49.43 | 67.59 | 94.35 | 110.69 |
| **11** | **1** | 61.47 | . | 61.47 | 61.47 | 61.47 | 61.47 | 61.47 |

GA: gestational age in weeks, n: number of patients, SD: standard deviation, Min: minimum value of serum progesterone in nmol/L, Max: maximum value of serum progesterone in nmol/L

Table S1D. Distribution of serum progesterone across gestation weeks 5 – 13 amongst women who presented with **threatened miscarriage and had a spontaneous miscarriage at or before 16 weeks [TMM]**

| **GA** | **n** | **Mean** | **SD** | **Min** | **10^th^ %** | **Median** | **90^th^ %** | **Max** |
| --- | --- | --- | --- | --- | --- | --- | --- | --- |
| **5** | **4** | 30.93 | 19.72 | 11.83 | 11.83 | 28.50 | 54.89 | 54.89 |
| **6** | **70** | 28.82 | 19.28 | 7.27 | 11.69 | 20.40 | 60.98 | 88.71 |
| **7** | **12** | 30.15 | 14.64 | 13.84 | 14.95 | 25.34 | 53.33 | 57.27 |
| **8** | **6** | 23.58 | 16.64 | 4.79 | 4.79 | 18.48 | 50.45 | 50.45 |
| **9** | **4** | 56.88 | 17.49 | 34.93 | 34.93 | 57.80 | 77.01 | 77.01 |
| **10** | **4** | 29.70 | 26.99 | 16.00 | 16.00 | 16.31 | 70.18 | 70.18 |

GA: gestational age in weeks, n: number of patients, SD: standard deviation, Min: minimum value of serum progesterone in nmol/L, Max: maximum value of serum progesterone in nmol/L
