# Supplementary material for: Serum progesterone distribution in normal pregnancies compared to pregnancies complicated by threatened miscarriage from 5 to 13 weeks gestation: a prospective cohort study
Source: BMC Pregnancy Childbirth. 2018 Sep 5;18:360. doi: 10.1186/s12884-018-2002-z (PMC6126027; doi:10.1186/s12884-018-2002-z)
Supplement: Supplementary file 2 — Figure S1A. Distribution of serum progesterone across gestation weeks 5 – 13 amongst women with low risk pregnancy [NP]. Figure S1B. Distribution of serum progesterone across gestation weeks 5 – 13 amongst women with threatened miscarriage [TM]. Figure S1C. Distribution of serum progesterone across gestation weeks 5 – 13 amongst women who presented with threatened miscarriage and had ongoing pregnancy at 16 weeks [TMO]. Figure S1D. Distribution of serum progesterone across gestation weeks 5 – 13 amongst women who presented with threatened miscarriage and had a spontaneous miscarriage at or before 16 weeks [TMM]. (ZIP 141 kb) [file 12884_2018_2002_MOESM2_ESM.zip › Supplemental Figure 1CR3.docx]

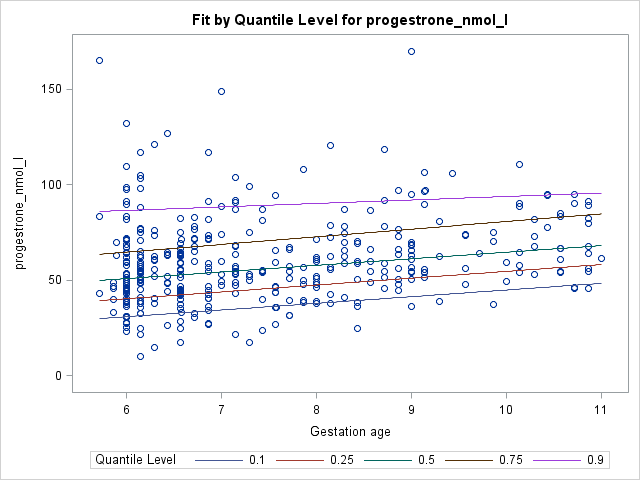


Distribution of serum progesterone amongst women with TM and ongoing pregnancy [TMO]

Serum progesterone (nmol/L)

Gestation age (weeks)
